# Factors Associated with Induced Abortion in Women Prostitutes in Asturias (Spain)

**DOI:** 10.1371/journal.pone.0002358

**Published:** 2008-06-04

**Authors:** Domingo Ojer Tsakiridu, Amalia Franco Vidal, Fernando Vázquez Valdés, Maria Luisa Junquera Llaneza, Jose Antonio Varela Uría, Mar Cuesta Rodríguez, Carmen López Sanchez, Margarita Busto Folgosa, Maria Jesús Fernández Ollero

**Affiliations:** 1 Centro de Salud de La Felguera, La Felguera, Voluntary of Médicos del Mundo (Doctors of the Word) Asturias, Asturias, Spain; 2 Servicio de Calidad, Hospital Universitario Central de Asturias, Oviedo, Asturias, Spain; 3 Servicio de Microbiología, Hospital Monte Naranco, Oviedo, Asturias, Spain; 4 Unidad de I.T.S., Hospital Monte Naranco, Oviedo, Asturias, Spain; 5 Unidad de I.T.S., Ambulatorio de Pumarín, Gijón, Asturias, Spain; 6 Médicos del Mundo (Doctors of the Word) Asturias, CEREDA, Oviedo, Asturias, Spain; Institute of Clinical Effectiveness and Health Policy, Argentina

## Abstract

**Background:**

The aim is to investigate the factors that might be associated with the presence of induced abortion (IA) in women prostitutes in Asturias (Spain).

**Methodology/Principal Findings:**

Cross-sectional descriptive study by self-completion questionnaire of 212 women prostitutes who attended the three Sexually Transmitted Disease Clinics in Asturias, between January–December 2003. The questionnaire was designed to investigate the women's perceived knowledge (what they claimed to know), their real knowledge (what they really knew), the use of contraceptive methods and socio-demographic variables. Multivariate analysis was carried out. 92% of the participants were immigrants. 76% were practising at brothel. 37.6% (95%CI:30.7–44.4%) reported to have undergone at least one IA during their life. According to the logistic regression the “presence of IA” was directly associated with the variables “number of pregnancies”(OR:65.82;95%IC:7.73–560.14) and “years of practising prostitution”(OR:1.13;95%CI:0.99–1.29); and inversely associated with “children”(0 = no children;1 = one or more children; OR:0.005;95%CI:0.000–0.057), “women's age”(OR:0.89;95%CI:0.82–0.97) and “real contraceptive knowledge”(OR:0.50; 95%CI:0.34–0.75). Married women were more likely to have undergone an IA (OR:2.74;95%IC:1.05–7.13). No association with “perceived contraceptive knowledge” was found.

**Conclusions/Significance:**

The characteristics more closely linked to the reproductive history of the women (such as “pregnancies”, “children”), together with the “real contraceptive knowledge” and the “time practising prostitution” explain the presence of IA better than factors more closely linked to the conditions in which the women practise prostitution (“place of activity”, “other activities compatible with prostitution”, “use of safe method in commercial relation”). It is possible that IA is being used as a birth control method, hypothesis suggested by the inverse association observed between the variable “children” and the “presence of IA”. Therefore, the promotion of the use of safe contraceptive methods should be a high-priority. If the real contraceptive knowledge was measured correctly, all strategies to increase it would be justified because it was inversely associated with the presence of IA.

## Introduction

The rates of induced abortion (IA) in Asturias in women between 15 and 44 years were the highest in Spain between 1991 and 1996 [1], [2], and although the situation has improved in recent years, Asturias still ranked fourth among the Spanish Autonomous Regions in 2000 [2]. This explains why the reduction of the number of unwanted pregnancies and IAs was one of the objectives detailed in the Health Strategies for Asturias [1], [3], [4] and in the last Health Plan covering the period 2004 to 2007 [4]. The development of sexual education and contraceptive programs was considered necessary for the achievement of this objective [1], [4].

There are very few studies published about the IA rates in women prostitutes, but it seems to be a very widespread problem amongst them: the only study in Europe was carried out in 1997 and showed that 33% of the women prostitutes in Rome had undergone an IA during the previous year [5]. Data from Cambodia, Korea and Gambia do not seem to be better [6], [7], [8], [9]. The only data available from Spain (from the 90s) also show high figures for IA: between 33% and 48% of women prostitutes had undergone at least one IA [10], [11], [12]. The only recent study in Spain with women was carried out only with immigrant women, and 30.5% stated that they had had one or more IAs during their life [13]. Other manuscripts also give high rates of abortion among women prostitutes, but they do not specify if they are only induced abortions [14], [15], [16]. In spite of all these data, neither the women's knowledge about contraceptive methods nor how they use them has been properly studied in women prostitutes, and there is no research that attempts to study the factors associated with the presence of IA. The public health studies have focussed on the incidence and prevalence of HIV and also on other Sexually-Transmitted Diseases (STD), due to their association with HIV, and on the use of condoms to prevent STD transmission [17], [18], since classically these women have been considered to constitute a high risk for the transmission of HIV and STD among the general population through their clients [19]. In Spain, the few studies that investigated issues related to knowledge about contraceptives, how they were used and IAs in this group of women were carried out in the 90s [11], [12], when the women who practised prostitution were in most cases Spanish. But the profile of these women has changed a lot in recent years due to the displacement of native women by immigrants, a phenomenon that has taken place not only in Spain but in the rest of Europe as well. It is very important to know and understand the factors associated with the presence of IAs in women prostitutes in order to plan any strategy, such as educational and contraceptive programs, aimed at reducing the incidence of IAs in this group of women.

Therefore, the aim of the present study is to investigate the socio-demographic factors and other factors related to the knowledge and use of contraceptive methods that might be associated with the presence of IA in women who practise prostitution in Asturias (Spain) and attended the STD Clinics of the Asturian Health Service and one maintained by Doctors of the World.

Finally, it should be pointed out that these clinics have become a reference point for women who practise prostitution in Asturias, thanks to the confidence that they have in the doctors and staff there. It is very important to remember that most of the women are irregular immigrants with no documentation, and because of this they do not use the standard community healthcare system. Through the clinics, nearly all their health problems are resolved, they receive contraceptive education and they are sent on to the corresponding specialists when their condition requires it.

## Methods

We implemented a descriptive cross-sectional study, by self-completion questionnaire (anonymous, but identifiable by means of a code). All the women were informed about the aim of the study, and then gave their verbal consent before being included in it. Participants did not receive any financial support. The research conformed to the Helsinki Declaration and to local legislation, and the Ethics Committee for Clinical Trials of Asturias (Comité de ética de ensayos clínicos de Asturias) approved it.

The sample size calculated for the worst case of IA (prevalence = 50%) by using the software package EPI INFO 6.04 was 215 people (desired precision 7%, rate of non-completion 10%). The study population was composed of women practising prostitution who had come to the STD centres of the Asturian Health Service (Monte Naranco Hospital or Pumarín Health Centre) and to the STD clinic of the PRIAPA Project for women prostitutes of Doctors of the World Asturias during the course of 2003 (N = 911). The exclusion criteria encompassed any women who after being informed about the study did not agree to participate, women with linguistic problems (the questionnaire was in Spanish) or others that prevented them from completing the questionnaire, and women at their first consultation (to avoid interfering with the incipient doctor-patient relationship). A consecutive sampling technique was used: we included the first two women who came to the above-mentioned clinics each day during the period of a year (January–December 2003) and who did not fulfil the exclusion criteria. The information was collected by means of a self-completion questionnaire presented to the women by nurses before or after the medical consultation. The validity of the questionnaire was guaranteed by an exhaustive bibliographical review that allowed the elaboration of an initial questionnaire, which was reviewed by a group of experts (doctors and nurses from the STD units who had been working with women prostitutes for more than 10 years, including the nurses who presented the questionnaire to the participants; one psychologist and one gynaecologist, both from the PRIAPA Project of Doctors of the World; one Public Health expert, who was qualified in sex education). Its viability and intelligibility was evaluated by a focus group discussion, consisting of 6 users of the Doctors of the World clinic who had not previously met each other (2 Colombians, 3 women from the Dominican Republic and a Spanish woman). Afterwards a pilot study (30 surveys) was carried out in the three clinics where the questionnaire was going to be employed. Finally, the definitive questionnaire form was reviewed after another meeting of the group of experts.

### Evaluated variables

*Socio-demographic variables*: “Age”, “Origin”, “Level of education”, “Marital status”, “Number of children”, “Number of pregnancies”.

*Variables related to prostitution*: “Period of time practising prostitution”, “Place where prostitution takes place”, “Other activities compatible with prostitution”.

*Variables related to IA*: “Number of pregnancy interruptions”, “Number of induced abortions” (this variable was measured by the question: “Of all the pregnancies terminated before they came to term, how many of them were interrupted voluntarily?”) “Age when they underwent their first IA”.

*Variables related to use of contraceptive method*: “Use of safe method in the last commercial vaginal relation” (considered as safe methods: condoms, oral contraceptives, intrauterine devices, diaphragm with spermicide; ligation; hormonal implants and intramuscular contraceptives).

*Variables related to the knowledge of contraceptive methods*:

- Contraceptive knowledge in general: “Perceived contraceptive knowledge” (or subjective contraceptive perception), measured by question number 1 of the annex; “Real contraceptive knowledge”, measured by four questions related to four different contraceptive methods (questions 3, 4, 5 and 6 of the “Appendix S1”).
- Knowledge of each individual contraceptive method: “Perceived knowledge (or subjective perception) of each individual method”: the methods studied are those that appear in question 2 of the annex; “Real knowledge of each individual method”: only the real knowledge about the four contraceptive methods mentioned above was evaluated (questions 3, 4, 5 and 6 of the “Appendix S1”).

A descriptive and bivariate analysis was carried out, with 95% confidence intervals and level of significance of 0.05. Bivariate analysis was used, according to the quantitative or qualitative nature of the variables (Chi-square, t student, Fisher's Exact Test, Mann-Whitney U, Correlation coefficients of Pearson or Spearman) and according to normality criteria (Kolmogorov Smirnov Z). A logistic regression was carried out, with 95% confidence intervals and level of significance of 0.05. The dependent variable was the “absence/presence of IA during all the life” of the women prostitutes. All the factors significantly associated with IA in the bivariate analysis were included in the logistic regression. There were also included other factors with no association in the bivariate analysis but which the group of experts and the research team thought might be associated with the dependent variable once the confounding factors were adjusted. The bivariate analysis and the logistic regression were carried out considering only the women who had at sometime been pregnant, because only this group of women, and not all women, had the possibility of having had an IA.

SPSS for Windows 10.0 software was used.

## Results

212 questionnaires were collected. The answer rate to each of the questions in the collected questionnaires was superior to 90%. The description of the socio-demographic variables and those related to the practice of prostitution are shown in Table 1.

**Table 1:** Socio-demographic characteristics and variables related to the practise of prostitution.

Variables	Subcategories	Value
Age		29.6 years (6.7)
Level of education:	-Primary incomplete	6.7% (14)
	-Primary completed	34.0% (71)
	-Secondary completed	51.6% (108)
	-Upper or university completed	7.7% (16)
Marital status:	-Single	55.5% (116)
	-Married or free union	27.3% (57)
	-Others	17.2% (36)
Number of children per women		1.2 (1.3)
Number of pregnancies per women		2.1 (1.8)
Women with one o more children		68.9% (146)
Women with one o more pregnancies (in all their life)		82.1% (174)
Women with two o more pregnancies		55.2% (117)
Origin:	-Latin Americans	82.4% (168)
	-Spanish	7.9 (16)
	-Others	9.8% (20)
Time practising prostitution		4.0 years (3.8)
Age at first practising prostitution		25.4 years (6.0)
Place of activity:	-I do not work at the moment	18.9% (39)
	-Club (brothel)	61.7% (127)
	-Contact flat	15.5% (32)
	-Street	1.5% (3)
	-Others	2.5% (5)
Other activities made compatible with prostitution:	-Only prostitution	55.6% (99)
	-Study	10.1% (18)
	-Cleaning	10.1% (18)
	-Domestic workers	7.9% (18)
	-Others	16.3% (29)

For quantitative data mean is indicated and standard deviation in brackets. For qualitative data, percentage, and absolute number in brackets.

Among all women, the percentage that reported having undergone at least one IA was 37.6% (95% CI: 30.7–44.4%), and when only the women who had at some point been pregnant were considered, this percentage was 46.1% (95% CI: 38.2–54.0). Among all women with one or more IAs during their life, the percentage who had undergone at least one IA before they started to practice prostitution was 69.7% (95% CI: 57.85–81.54), and the percentage who had undergone at least one IA during their prostitution life was 54.0% (95% CI: 39.2–68.8).

61.2% (95% IC: 54.2–68.1) claimed to have sufficient information about contraceptive methods (perceived contraceptive knowledge); 26.4% (95% IC: 20.2–32.6) had acceptable real knowledge of methods, that is to say, gave valid answer to questions 3, 4, 5 and 6 of the “Appendix S1”. The average number of questions they answered correctly was 2.52 (95% IC: 2.36–2.68); 97.6% (95% IC: 94.6–99.2) used a safe method in the last commercial relation and 79.6% (95% IC:73.6–85.6) used a safe method in the last private relation.

In the bivariate analysis carried out only with the women who had at some time been pregnant, five factors were significantly associated with IA when considering women with at least one IA versus women with no IA during all their life: the “number of pregnancies”, the “number of children”, the “real knowledge” (not the “perceived knowledge”), the “use of safe method in the last commercial relation” and the “origin”. The mean “number of pregnancies” among the women with one or more IAs during their life was higher than in the group with no IA (p<0.001, Table 2), and the mean “number of children” was lower (p = 0.033, Table 2). The percentage of women who had acceptable real knowledge of contraceptive methods was twice as high in the group of women with no IA than in the group with one or more IAs (p = 0.033, Table 3). The percentage of women who “used a safe method in the last commercial relation” was higher in the group with no IA than in the group with one or more IAs (p = 0.042, Table 3). And the percentage of immigrant women is higher in the group with IA than in the group with no IA (p = 0.040, Table 2).

**Table 2:** Socio-demographic variables and variables related to prostitution in women, according their history of IA.

Variables	Subcategories	Women with one or more IAs during their life	Women with no IA during their life	“p-value”
**Women's Age** (mean)		30.1 (28.5–31.7)	30.8 (29.4–32.3)	0.398
**Pregnancies** (mean)		3.2 (2.7–3.6)	2.1 (1.8–2.4)	**<0.001**
**Children** (mean)		1.3 (1.0–1.6)	1.7 (1.4–2.0)	**0.033**
**Years practising prostitution** (mean)		4.6 (3.6–5.6)	3.7 (3.0–4.6)	0.242
**Women with two o more pregnancies**		83.8% (74.7–92.9)	58.4% (47.7–69.3)	**<0.001**
**Women with one o more children**		71.4% (60.7–82.2)	94.4% (87.5–98.2)	**<0.001**
**Education level:**	-Primary incomplete or completed	40.8% (29.1–52.5)	42.7% (31.9–53.3)	0.965
	-Secondary completed	52.6% (40.7–64.5)	50.6% (39.6–61.6)	
	-Higher or university completed	6.6% (2.2–14.7)	6.7% (1.0–12.5)	
**Marital status**	-Married or free union	36.0% (24.5–47.5)	25.6% (16.0–35.1)	0.146
	-Single	64.0% (52.5–75.5)	74.0% (64.9–84.0)	
**Origin**	-No Spanish	97.3% (90.4–99.7)	88.8% (81.6–95.9)	**0.040**
**Place of activity**	-Club (brothel)	83.1% (72.6–93.5)	73.0% (62.2–83.8)	0.167
**Others activities made compatible with prostitution**	-Only prostitution	58.5% (45.7–71.2)	53.3% (41.4–65.3)	0.542
	-Other activities besides prostitution	41.5% (28.8–54.3)	46.7% (34.7–58.6)	

95% CI in brackets.

**Table 3:** Contraceptive knowledge and use of safe method in women, according their history of IA.

Variables	Subcategories	Women with one or more IAs during their life	Women with no IA during their life	“p-value”
**Perceived knowledge:**	-%women who claim to have sufficient information about contraceptive methods	61.1 (49.1–73.1)	58.8 (47.8–69.9)	0.771
**Real knowledge:**	-% women who have acceptable real knowledge of contraceptive methods (valid answers to the 4 questions)	16.9 (7.9–25.9)	31.1 (30.0–41.2)	**0.033**
	-Average number of questions with valid answers in relation to the 4 contraceptive methods evaluated	2.13 (1.85–2.41)	2.71 (2.48–2.94)	0.002
**Use of safe method:**	-%women who use a safe method in the last commercial relation	94.7 (87.1–98.5)	99.2 (95.9–99.9)	**0.042**
	-%women who use a safe method in the last private relation	75.4 (64.5–86.2)	82.7 (73.9–91.6)	0.268

95% CI in brackets.

When a logistic regression was carried out considering only the women who had at some time been pregnant during their life, and using the “absence/presence of IA during all the life” as dependent variable (Table 4), a direct and statistically significant association was found between the “presence of IA” and the “number of pregnancies” categorized in two categories: one pregnancy, and two o more (OR: 65.82; 95%CI 7.73–560.14). And a direct and almost statistically significant association was also found with the “years of practising prostitution” (“p value”: 0.072; OR: 1.13; 95%CI 0.99–1.29). Women with a steady partner were more likely to have suffered an IA (OR: 2.74; 95%IC: 1.05–7.13). We found an inverse and statistically significant association between the “presence of IA” and three variables: “real contraceptive knowledge” (OR: 0.50; 95%CI: 0.34–0.75), “women's age” (OR: 0.89; 95%CI: 0.82–0.97) and “children” categorized in two categories: no children, and some child (OR: 0.005; 95%CI: 0.000–0.057). “Perceived contraceptive knowledge” was not significantly associated with “presence of IA”.

**Table 4:** Logistic regression to detect association between different variables and induced abortion.

Variables	B	S.E	Odds Ratio (95%CI)	“p-value”
Real contraceptive knowledge (number of questions with valid answers)	−0.68	0.20	0.50 (0.34–0.75)	**0.001**
Number of pregnancies (0 = one pregnancy; 1 = two or more)	4.19	1.09	65.82 (7.73–560.14)	**<0.001**
Children (0 = no children; 1 = one o more children)	−5.29	1.24	0.005 (0.000–0.057)	**<0.001**
Women's age (years)	−0.12	0.04	0.89 (0.82–0.97)	**0.006**
Average time practising prostitution (years)	0.12	0.07	1.13 (0.99–1.29)	0.072
Marital status (0 = single; 1 = married or free union)	1.01	0.49	2.74 (1.05–7.13)	**0.038**

95% CI in brackets.

B: Coefficient of logistic regression.

S.E: Standard Error.

Naglekerke R square = 0.53; 0 = No IA during all their life; 1 = IA during all their life.

## Discussion

It was a priority to guarantee the anonymity and the confidentiality of the respondents since many of the women who practise prostitution are in an irregular legal situation in Spain (many are immigrants “without papers”) and since the subject of IA is particularly delicate for them (IA is illegal in Spain). According to authors' data we know most immigrant women prostitutes in our region are in an “irregular situation” (María Jesús Fernandez Ollero is at this moment conducting the first study about the characteristics of women prostitutes that has been carried out in Asturias since 1992, commissioned by the *“Consejería de Vivienda y Bienestar Social”* of the Asturian Government). For these reasons we preferred an anonymous self-completion questionnaire: this type of questionnaire would make the answers less embarrassing for the participants, would guarantee better confidentiality and anonymity, and would also diminish the social convenience bias (the participants did not need to admit that some of their sexual behaviour was not what had been recommended in the STD clinic).

In spite of the delicate subjects dealt with and the group of the population studied the answer rate for each question is acceptable, possibly because of the women's confidence in the STD clinic personnel and because the questionnaire was of the self-completion type.

The most important limitations were related to the sample representativeness, since women who had difficulty with the written language and non-Spanish speakers (the questionnaire was written, self-completion, and in Spanish) and women who practise prostitution in the street (it is known that they rarely go to the STD clinics) might be under-represented. However, according to the study of María Jesús Fernandez Ollero mentioned before, we know that these under-represented sub-groups are a minority in relation to all women who practise prostitution in our region. Furthermore, we verified that the distribution by countries of origin of the women who formed our study sample was very similar to that of all the women who had come to the three STD clinics during the previous twelve months of data collection. We also verified that neither was there any significant difference regarding the origin (Latin Americans, Spanish and others) and place of activity (club, contact flat and street) between our sample (212 women) and the whole population our sample was drawn from (the 911 women who practice prostitution and had come to the STD centres during 2003). Nonetheless we should not forget that our population may not be representative of all women who practise prostitution in Asturias (according to the provisional data of Maria Jesús Fernandez Ollero, there could be between 1300 and 1400 women practising prostitution in our region), because the women who attend STD clinics go there spontaneously, and there may exist personal characteristics that condition their attendance at these clinics and which differentiate them from the group that does not attend. Another major concern is about the internal validity of the results, due to the lack of specific questionnaires about IA for women prostitutes, and because the validation of questionnaires is very complex in this social group.

Finally it is necessary to indicate that any study that attempts to take a quantitative approach to IA cannot ignore a limitation with respect to the concept and measurement of IA. It is necessary to assume a possible miss-classification bias which arises if a woman declares an abortion to be spontaneous when it is really induced (and this tendency could be more accentuated when the survey is carried out in a country where IA is penalized, as it is in Spain, and when women practising prostitution are already stigmatised). In some studies designed to determine the prevalence of IA by questionnaires before or after an intervention [20], or which try to study variables associated with IA in the general population [21], this limitation is controlled by considering the number of abortions in general (voluntary and spontaneous) as an index of IA. In our study the percentage of spontaneous abortions in relation to total pregnancies was 11.03%, very close to that found in the general population (we can assume 10–15% from literature) [22], [23]. For this reason it appears that if there has been a miss-classification bias reducing the number of induced abortions in favour of spontaneous ones, the bias is small.

It is important to mention that because of our methodology (cross-sectional descriptive study), the time sequence is not guaranteed, and so we can only conclude the presence or absence of association between the studied variables and IA. The intention of the study is not to verify hypotheses that could explain the associations found, but only to formulate them. The longitudinal studies that could verify these hypotheses are very difficult to carry out in women who practise prostitution, because of the continuous geographic mobility that characterizes this social group, a mobility due, above all, to the fact that almost all of them are immigrants.

The figures for IA we found are high, but although there are very few data available in PubMed and Embase on women prostitutes in other countries, our figures are very similar to those reported in those studies [5]–[8], [15], [16].

It is significant to point out that we did not find any articles in PubMed o Embase that studied factors associated with IA among women prostitutes, for which reason our findings could unfortunately not be compared with others.

In the logistic regression (carried out considering only the women who had at some time been pregnant, Table 4), the variable “number of pregnancies” was directly associated with the “presence of IA” (the higher the number of pregnancies, the higher the probability of having undergone one o more IAs: the women with two o more pregnancies were more likely to have suffered an IA than women with only one pregnancy). The variable “children” was inversely associated with the “presence of IA” (the women with one or more children were less likely to have suffered an IA than the women with no children, that is to say, the woman with no children were more likely to have suffered an IA). This second (and inverse) association, together with the high figures for IA found in the women prostitutes in our study, allows us to formulate the hypothesis that IA could be being used as a birth control method, a hypothesis suggested by other investigators [24], [25], [26] who studied this phenomenon in women from the general population in certain developing countries. Therefore, if this finding is confirmed, it would be a priority to promote the use of safe contraceptive methods that guarantee better birth control.

In the logistic regression (Table 4), “women's age” was inversely associated with the presence of IA, so, the lower their age, the higher the probability of having undergone an IA. This association was not found in the bivariate analysis (Table 2), maybe because the variable “number of pregnancies” is behaving as a confounding factor (the older a women is, the higher the probably of her having been pregnant, and therefore, the higher the probability of her having undergone an IA). In the logistic regression the confounding factor “number of pregnancies” is adjusted (and also other confounding factors), and so an inverse relationship between “women's age” and “presence of IA” is obtained (Table 4): given the results of the logistic regression it could be said that with the same number of pregnancies, the younger women are more likely to have undergone an IA. The relationship between the “women's age” and IA is consistent with the findings in other studies carried out in the general population (not women prostitutes), in which after adjusting the number of previous pregnancies, the probability of having undergone one or more IAs is found to be higher in younger women [27].

Although the variable “time practicing prostitution” was almost significantly associated with the “presence of IA” (Table 4), the interpretation of this association is not clear: the proportion of women who had undergone one or more IAs before they started to practise prostitution was very high. Therefore, and since the data are not conclusive, it would be of interest to repeat the study in an immigrant population residing in Asturias which does not practise prostitution, in order to better evaluate the role that the practise of prostitution is playing in our IA figures, comparing the IA figures for the immigrant women who do not practise prostitution with those of the prostitute and immigrant women included in our study. Only 1.7% of women in the general female population in Spain reported an IA during all their life [28].

“Marital Status” was significantly associated with the “presence of IA”. According to our study, women with a steady partner are associated with a higher probability of IA than single women. What perhaps would be of interest is a qualitative investigation to enable women to express the role their private partners might have played in decisions about abortion and those related to having children.

“Real contraceptive knowledge” was inversely associated with the “presence of IA”: the less the women's knowledge, the higher was the probability of their having undergone an IA. Although it is well known that knowledge is not sufficient in itself to increase the use of contraceptives methods [29], [30], [31] and in our study the “real contraceptive knowledge” has not been associated with the “use of safe method in the last relation” (results not shown), the relationship between real knowledge and IA is coherent. It is logical to suppose that when a woman who really knows how a contraceptive works uses that contraceptive, she uses it correctly, and so the contraceptive is more effective in preventing unwanted pregnancies (although higher real knowledge does not mean greater use of contraceptive methods). In any case, finding this association between real knowledge and the absence/presence of IA supports the notion that the questionnaire used in the study was able to achieve an approximation of what it was designed to measure (real contraceptive knowledge), because if it had not, it is very unlikely that this significant association would have been apparent. Therefore, if those findings are right, all the strategies intended to increase the real knowledge the women prostitutes have about contraceptive methods are justified, since they could have an influence on the absence/presence of IAs in this group of women.

It was no found a statistically significant association between the “use of a safe method in the last commercial relation” and the “presence of IA” in the logistic regression. It could simply be because this association really does not exist, or because the proportion of women who did not use a safe method in their last commercial relation was so low that it did not allow the detection of a significant association although such an association may exist. In our study, as in others [32], the attitude towards contraception in the last relation was used as an indication of the habitual attitude towards contraception, because the latter is a much more difficult variable to measure than the former.

The variables used in the logistic regression seem to be the appropriate variables for the analysis, because they explain the phenomenon we are studying to a high degree (Naglekerke R square is very high), and all of the variables used in the analysis were significantly (or almost significantly) associated with the “absence/presence of IA” (Table 4). Also, the results were very coherent, as we have explained in the discussion section.

The results of the study show that the characteristics most closely linked to the reproductive history of the women prostitutes (such as “pregnancies”, “children”, “marital status”), together with the “real contraceptive knowledge” and the “time practising prostitution” explain the presence of IA better than the factors more closely related to the conditions in which the women practise prostitution (“place of activity”, “other activities compatible with prostitution”, “use of safe method in commercial relation”, etc). So, we think that the factors most closely related to reproductive history (and therefore, linked to socio-cultural aspects and probably shared by women from the general population of the countries of origin of our participants), together with the practice of an activity such as prostitution, with a high risk of unwanted pregnancies, perhaps could explain the high number of IAs we have found in our study. IA may be being used as a birth control method, a hypothesis that is suggested by the inverse association that was observed between the variable “children” and the “presence of IA”. Therefore, the promotion of the use of safe contraceptive methods for commercial relations should be a high-priority. If the real contraceptive knowledge of the women has been measured correctly, all strategies to increase it would be justified because it was inversely associated with the presence of IA. Nevertheless, the only way of ensuring that increased contraceptive knowledge has an impact on abortion rates would be to design a randomised controlled trial. Qualitative research to better understand the significance of IA in this group of women should also be considered for future studies.

## Supporting Information

Appendix S1 (0.07 MB DOC)
