# Supplementary material for: Factors Associated with Induced Abortion in Women Prostitutes in Asturias (Spain)
Source: PLoS One. 2008 Jun 4;3(6):e2358. doi: 10.1371/journal.pone.0002358 (PMC2396519; doi:10.1371/journal.pone.0002358)
Supplement: Appendix S1 — (0.07 MB DOC) [file pone.0002358.s001.doc]

**Appendix S1**

Next we show the 6 questions that were used in the self-completion questionnaire to study the knowledge of contraceptive methods, real and perceived. The original questions, just as they were in the questionnaire, are presented first and afterwards an English translation to facilitate comprehension, although only the original version was obtained according to the process described in “methods”:

Original version:

1.- ¿Tienes suficiente información acerca de los métodos anticonceptivos?

| SÍ |  | NO |  | NO SÉ |
| --- | --- | --- | --- | --- |

2.- De los siguientes métodos anticonceptivos, señala cuáles sabes utilizar y cuáles no:

| a.- Condón, preservativo o gomas ……………………….. | Sé usarlo |  | No sé usarlo |  |
| --- | --- | --- | --- | --- |
| b.- La píldora, pastas o anticonceptivos orales… | Sé usarlo |  | No sé usarlo |  |
| c.- La T, DIU, o espiral ………………………………………. | Sé usarlo |  | No sé usarlo |  |
| d.- Óvulos o cremas espermicidas ……………………. | Sé usarlo |  | No sé usarlo |  |
| e.- Ligadura de trompas ……………………………………… | Sé usarlo |  | No sé usarlo |  |
| f.- El diafragma ……………………………………………………. | Sé usarlo |  | No sé usarlo |  |
| g.- La marcha atrás o el coito interrumpido ….. | Sé usarlo |  | No sé usarlo |  |
| h.- La píldora del día siguiente …………………………. | Sé usarlo |  | No sé usarlo |  |
| i.- La inyección o topasel ……………………………………. | Sé usarlo |  | No sé usarlo |  |

| j.- Otros. Citar |  |
| --- | --- |

3.- –¿El condón es seguro para evitar el embarazo y las enfermedades de transmisión sexual si se coloca justo en el momento de eyacular o de correrse?

| SÍ |  | NO |  | NO SÉ |
| --- | --- | --- | --- | --- |

4.- –¿Si me olvido de tomar la píldora más de un día me puedo quedar embarazada?

| SÍ |  | NO |  | NO SÉ |
| --- | --- | --- | --- | --- |

5.- -¿La marcha atrás o coito interrumpido es un método muy eficaz para evitar el embarazo si el hombre se controla bien?

| SÍ |  | NO |  | NO SÉ |
| --- | --- | --- | --- | --- |

6.- –¿La T o DIU me la tiene que poner un médico?

| SÍ |  | NO |  | NO SÉ |
| --- | --- | --- | --- | --- |

English translation:

1.- Do you have enough information about contraceptive methods?

| YES |  | NO |  | I DON´T KNOW |
| --- | --- | --- | --- | --- |

2.- From the following contraceptive methods, indicate to us which ones you know how to use and which you don't:

| a.- Condoms ……………………….. …………………………………… | I know how to use it |  | I don't |  |
| --- | --- | --- | --- | --- |
| b.- Contraceptive pills, ………………………………………….. | I know |  | I don't |  |
| c.- DIU …………………………………………………………………….. | I know |  | I don't |  |
| d.- Spermicide creams.............................................. | I know |  | I don't |  |
| e.- Tubal sterilization, ligation ……………………………. | I know |  | I don't |  |
| f.- Diaphragm ………………………………………………………….. | I know |  | I don't |  |
| g.- Coitus interruptus……………………………………………… | I know |  | I don't |  |
| h.- The day after pill ……………………………………………... | I know |  | I don't |  |
| i.- Injection, topasel ………………………………………………. | I know |  | I don't |  |
| | j.- Other methods. Please, tell us |  | | --- | --- | |  |  | | |
|  |  | | | |

3.- Is the condom safe for avoiding pregnancy and sexually transmitted diseases, if he puts it on just before he comes?

| YES |  | NO |  | I DON'T KNOW |
| --- | --- | --- | --- | --- |

4.- When I forget to take the pill for longer than a day, may I get pregnant?

| YES |  | NO |  | I DON'T KNOW |
| --- | --- | --- | --- | --- |

5.- Is coming outside (coitus interruptus) a safe method to avoid pregnancy, when he does it properly?

| YES |  | NO |  | I DON'T KNOW |
| --- | --- | --- | --- | --- |

6.- Do I have to get the DIU put in me by a doctor?

| YES |  | NO |  | I DON'T KNOW |
| --- | --- | --- | --- | --- |
